# Effect of commercial breakfast fibre cereals compared with corn flakes on postprandial blood glucose, gastric emptying and satiety in healthy subjects: a randomized blinded crossover trial

**DOI:** 10.1186/1475-2891-6-22

**Published:** 2007-09-17

**Authors:** Joanna Hlebowicz, Jennie Wickenberg, Rickard Fahlström, Ola Björgell, Lars-Olof Almér, Gassan Darwiche

**Affiliations:** 1 Department of Medicine, University of Lund, Malmö University Hospital, Malmö, Sweden; 2 Department of Radiology, University of Lund, Malmö University Hospital, Malmö, Sweden; 3 Medical School, University of Lund, Malmö University Hospital, Malmö, Sweden

## Abstract

**Background:**

Dietary fibre food intake is related to a reduced risk of developing diabetes mellitus. However, the mechanism of this effect is still not clear. The aim of this study was to evaluate the effect of commercial fibre cereals on the rate of gastric emptying, postprandial glucose response and satiety in healthy subjects.

**Methods:**

Gastric emptying rate (GER) was measured by standardized real time ultrasonography. Twelve healthy subjects were assessed using a randomized crossover blinded trial. The subjects were examined after an 8 hour fast and after assessment of normal fasting blood glucose level. Satiety scores were estimated and blood glucose measurements were taken before and at 0, 20, 30, 40, 60, 80, 100 and 120 min after the end of the meal. GER was calculated as the percentage change in the antral cross-sectional area 15 and 90 min after ingestion of sour milk with corn flakes (GER1), cereal bran flakes (GER2) or wholemeal oat flakes (GER3).

**Results:**

The median value was, respectively, 42% for GER1, 33 % for GER2 and 51% for GER3. The difference between the GER after ingestion of bran flakes compared to wholemeal oat flakes was statistically significant (p = 0.023). The postprandial delta blood glucose level was statistically significantly lower at 40 min (p = 0.045) and 120 min (p = 0.023) after the cereal bran flakes meal. There was no statistical significance between the areas under the curve (AUCs) of the cereals as far as blood glucose and satiety were concerned.

**Conclusion:**

The result of this study demonstrates that the intake of either bran flakes or wholemeal oat flakes has no effect on the total postprandial blood glucose response or satiety when compared to corn flakes. However, the study does show that the intake of cereal bran flakes slows the GER when compared to oat flakes and corn flakes, probably due to a higher fibre content. Since these products do not differ in terms of glucose response and satiety on healthy subjects, they should be considered equivalent in this respect.

**Trial registration:**

ISRCTN90535566

## Background

In Sweden and worldwide the incidence of type 2 diabetes mellitus is increasing rapidly. To prevent the development of diabetes mellitus, the American Diabetes Association recommends a reduction in caloric intake and increased consumption of dietary fibre and food containing whole grain [1]. An increased intake of fibre has been shown to reduce the risk of diabetes [2,3]. Whether low-glycemic-index food in fact prevents diabetes mellitus is still unclear [2,4-7]. However, a low-glycemic-index diet that reduces postprandial hyperglycemia is recommended by the American Diabetes Association (ADA) to control glycemia in patients with diabetes [8,9].

Fibre has been shown to delay gastric emptying rate, reduce the glycemic response and delay the return of hunger in healthy subjects [10]. It has been assumed that fibre fermented in the colon by the bacterial flora releases short chain fatty acids, thus lowering postprandial glucose levels [11-14]. Moreover, this fermentation has shown to result in a suppression of the hepatic glucose production and serum-free fatty acids [15]. Colonic fermentation, measured by breath hydrogen test, has been observed – after a meal consisting of ingestible carbohydrates – to reduce the insulin and glucose response at the following meal. This effect is called a second meal effect. [16]. However, another study showed that meals with what was assumed to be fermentable carbohydrates did not improve glucose or insulin response at the second meal [17]. A recently published study shows that an increased 3-day intake of insoluble fibre in obese subjects improved whole-body insulin sensitivity [18].

The β-glucan effect is not fully understood. Products enriched with β-glucan have been shown to reduce postprandial glucose and insulinemic responses in healthy subjects [19-21] and in type 2 diabetes patients [22-24]. Reduced postprandial glucose and insulin concentrations after consumption of viscous types of fibre have been discussed to be caused by delayed mouth-to caecum transit and delayed absorption of glucose in the small intestine [25]. The viscosity of oat gum, an oat extract composed of β-glucan, has been shown to cause a reduction in plasma glucose and insulin levels [26]. However, lower postprandial glucose and insulin concentrations have not been shown to be caused by the fermentation of β-glucan in the colon [27]. Another mechanism discussed is that β-glucan delays gastric emptying.

Healthy subjects are recommended to consume products with fibre to prevent the development of diabetes mellitus. Also, patients with diabetes consume commercial products with fibre and low glycemic food to control the blood glucose levels. This study was designed to determine whether there is a delay in gastric emptying in healthy subjects, thus affecting postprandial blood glucose levels and satiety, after consumption of commercially popular fibre cereals.

## Methods

Twelve healthy subjects (six men and six women; mean age 28 ± 4 years [range 23–36 years]; mean BMI 22 ± 2 kg/m^2 ^[range 19–24 kg/m^2^], without symptoms or a prior history of gastrointestinal disease, abdominal surgery or diabetes mellitus were included in the study. One subject had been appendectomized. None of the subjects used any drugs on the examination day. Three of the women, including one with polycystic ovary syndrome, had birth control medication. The subject with the polycystic ovary syndrome had a BMI 21 kg/m^2 ^and previously underwent a glucose tolerance test which proved normal. All subjects were recruited from the population of a southern county of Sweden. Four of the subjects were smokers and two were snuff users. The subjects were examined between 8:00 and 10:00 am after an 8 hour fast. Smoking and snuff-taking were prohibited for 8 h before the test as well as during the test. Each subject was checked for normal fasting blood glucose concentration on the day of the examination. For subjects with symptoms from the gastrointestinal tract (diarrhoea or constipation) on the examination day, the examination was postponed. The test meals consisted of 300 g sour milk (Skånemejerier, 205 03 Malmö, Sweden) (caloric value 135 kcal) and 50 g cereal bran flakes (Kellogg's All-Bran, Kellogg's, Sverige Konsumentkontakt, Box 742, 194 27 Upplands Väsby, Sweden) (caloric value 163 kcal) or wholemeal oat flakes (Frebaco Fullkorns Havreringar, Frebaco Kvarn AB, Box 878, 531 18 Lidköping, Sweden) (caloric value 185 kcal). The reference meal, consisting of 50 g corn flakes (Kellogg's Corn Flakes, Nordisk Kellogg's, Sverige Konsumentkontakt, Box 742, 194 27 Upplands Väsby, Sweden) (caloric value 185 kcal), had the same brand and quantity of sour milk as the test meal (Table 1). The meals were served in a random order more than one week apart. Each meal was ingested within 5 minutes.

**Table 1:** Nutrient composition of the test product portions.

	Sour Milk	Frebaco Wholemeal Oatflakes	Kellogg's All-Bran Regular Flakes	Kellogg's Cornflakes
	300 g	50 g	50 g	50 g

Total energy (kcal)	135	185	163	185
Total protein (g)	12	6	5	3.5
Total fat (g)	1.5	2	1	0.35
Total Carbohydrate (g)	18	35.5	33.5	42
Sugar (g)	15	0.75	11	4
Total Fibre (g)		4	7.5	1.5
β-glucan (g)		0.5		

GER was estimated using a previously described standardised ultrasound method [28]. The sonographic examination was performed using two different ultrasound machines (Siemens Acuson Sequoa 512 and Aloka Prof. Sound) with an abdominal transducer multi-MHz. For every single calculation of GER the same machine was used. The measurements of the gastric antrum were performed by the same radiologist who was blinded with regard to the meals. At each observation of the gastric antrum the abdominal aorta and the left lobe of the liver were used as internal landmarks. The subjects were examined lying down, but they were in upright position between examinations. Measurements were taken 15 and 90 min after the end of meal ingestion. Gastric emptying was expressed as the percentage change of the antral cross-sectional area from 15 to 90 min. At each examination three measurements of the longitudinal (d1) and anteroposterior (d2) diameters were performed and mean values were used to calculate the cross-section area of the gastric antrum using the following formula:

Antrum area = π× r^2 ^= π× d1/2 × d2/2 = π× d1 × d2/4

The gastric emptying (GER) was calculated using the following formula:

GER = [1- (Antrum area 90 min/Antrum area 15 min)] × 100

Finger-prick capillary samples were taken before and at 0, 20, 30, 40, 60, 80, 100 and 120 min after the end of the meal to measure glucose. Blood glucose concentrations were measured with HemoCue Glucose system (HemoCue AB, Ängelholm, Sweden). A validated satiety score numerical scale was used according to the method of Haber et al on the basis of a scoring system with grades from -10 cm (extreme hunger) to 10 cm (extreme satiety) [29]. Satiety score was estimated before the meal and at 0, 20, 30, 40, 60, 80, 100 and 120 min after the end of the meal.

All subjects provided written informed consent. The study was approved by the Ethics Committee at Lund University and performed according to the Helsinki Declaration.

Median values with quartiles (q1 to q3) are presented for the antral cross-sectional areas and the GER. Delta values of blood glucose and satiety scores are calculated as changes at 0, 20, 30, 40, 60, 80, 100 and 120 min after the end of the meal from a fasting value. The AUCs for each subject were determined for the delta blood glucose and satiety (Graph Pad PRISM, version 4, San Diego). The AUCs were calculated above zero. The AUCs values are presented as means ± SEMs. All statistical calculations were performed in SPSS for Windows. Significant differences of GER, gastric antral cross-sectional areas, delta blood glucose and AUCs were evaluated with the Wilcoxon t-test. Values of P < 0.05 were considered significant.

## Results

### Postprandial blood glucose response

**Figure 1:**
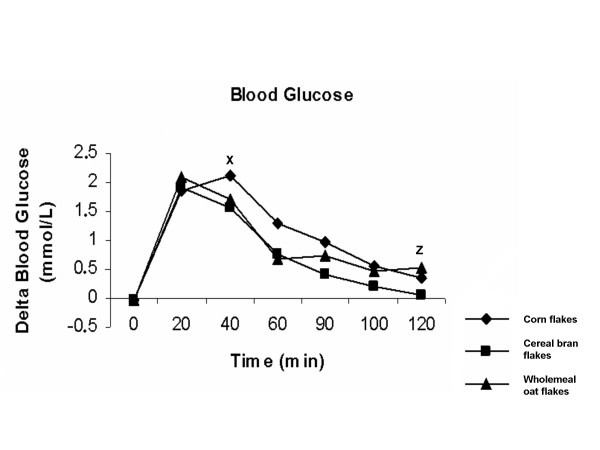
Means (± SEM) incremental blood glucose concentrations in twelve healthy subjects after ingesting meals consisting of sour milk with cereal bran flakes, corn flakes or wholemeal oat flakes. Significant differences calculated with the Wilcoxon t-test. X Cereal bran flakes significantly different in response compared to corn flakes (p < 0.05). Z Cereal bran flakes significantly different from response compared to wholemeal oat flakes (p < 0.05).

Ingestion of cereal bran flakes resulted in a significantly lower blood glucose response in the initial postprandial phase (40 min) than did the reference meal of corn flakes (p = 0.045) (Figure 1). Ingestion of cereal bran flakes resulted in a significantly lower blood glucose response in the late postprandial phase (120 min) than did wholemeal oat flakes (p = 0.023) (Figure 1). However, the blood glucose AUCs did not differ significantly between cereal bran flakes, wholemeal oat flakes and corn flakes (Table 2).

**Table 2:** Postprandial blood glucose areas under the curve (AUCs) after ingestion of meals consisting of wholemeal oat flakes, cereal bran flakes or corn flakes in twelve healthy subjects^1^. Significant differences of postprandial blood glucose AUCs were calculated with the Wilcoxon t-test. There were no significant differences between the AUCs.

AUC	Wholemeal Oat Flakes mmol * min/L	Cereal Bran Flakes mmol * min/L	Corn Flakes mmol * min/L
0 – 5 min	0.3 ± 0.1	1.3 ± 0.1	0.3 ± 0.1
0 – 25 min	21.2 ± 2.8	19.3 ± 1.4	19.3 ± 3.4
0 – 45 min	58.9 ± 7.1	53.8 ± 3.9	59.2 ± 10.2
0 – 65 min	83.0 ± 12.4	76.9 ± 7.7	93.8 ± 16.9
0 – 85 min	97.8 ± 16.3	88.7 ± 9.9	116.8 ± 21.8
0–105 min	110.1 ± 18.9	96.0 ± 10.4	124.4 ± 26.4
0 – 125 min	120.6 ± 21.5	106.8 ± 12.9	143.0 ± 26.3

^1^Mean ± SEM; n = 12

### Gastric emptying rate

**Figure 2:**
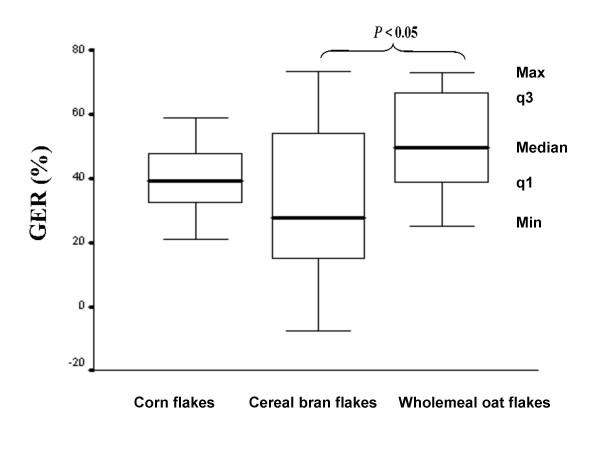
Gastric emptying of sour milk with cereal bran flakes, wholemeal oat flakes or corn flakes, estimated as gastric emptying rate (GER), in twelve healthy subjects. The median, minimum (Min), and maximum (Max) values and the values of the first (q1) and the third (q3) quartiles are shown. Significant differences were calculated with the Wilcoxon t-test. Cereal bran flakes significantly different in response compared to wholemeal oat flakes (p < 0.05)

The median values of the antral cross-sectional area after ingestion of the cereal bran flakes meal were 641 ± 197 mm^2 ^(range 418 to 1035 mm^2^) (q1 = 524 mm^2^, q3 = 824 mm^2^) and 331 ± 253 mm^2 ^(range 137 to 924 mm^2^) at 15 and 90 min, respectively, after the end of the meal. In the same subjects the median values of the antral cross-sectional area after the ingestion of the wholemeal oat flakes meal were 743 ± 240 mm^2 ^(range 498 to 1188 mm^2^) (q1 = 568 mm^2^, q3 = 1003 mm^2^) and 331 ± 226 mm^2 ^(range 149 to 852 mm^2^) (q1 = 205 mm^2^, q3 = 626 mm^2^) at 15 and 90 min after the end of the meal. In the same subjects the median values of the antral cross-sectional area after the ingestion of the corn flakes meal were 716 ± 187 mm^2 ^(range 170 to 740 mm^2^) (q1 = 436 mm^2^, q3 = 905 mm^2^) and 481 ± 227 mm^2 ^(range 380 to 1008 mm^2^) (q1 = 251 mm^2^, q3 = 495 mm^2^) at 15 and 90 min after the end of the meal. There were no significant differences between gastric antral cross-sectional at 15 or 90 min between the different meals. The median value of GER after the cereal bran flakes meal was estimated at 28% (range -8% to 73%) (q1 = 15%, q3 = 56%) compared to the median value of GER after the wholemeal oat flakes meal which was estimated at 50 % (range 25% to 73 %) (q1 = 38%, q3 = 70%). The median value of GER after the corn flakes meal was estimated at 39 % (range 21% to 73 %) (q1 = 31%, q3 = 49%). The cereal bran flakes meal had a significantly lower GER compared to wholemeal oat flakes meal (p = 0.023) (Figure 2). There were no significant differences between cereal bran flakes or wholemeal oat flakes compared to corn flakes with regard to GERs (Figure 2).

### Satiety

**Figure 3:**
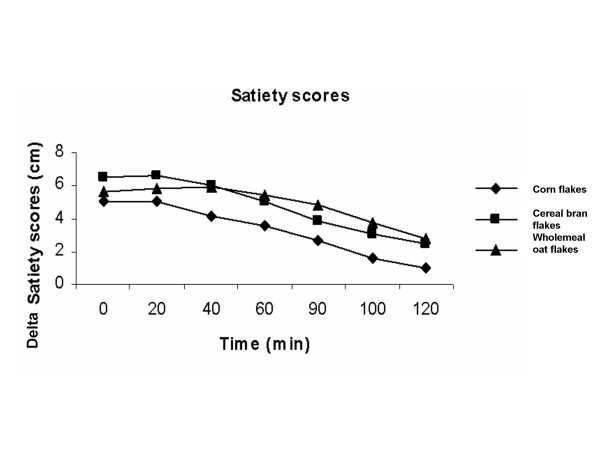
Means (± SEM) incremental satiety scores in twelve healthy subjects after ingesting meals consisting of sour milk with cereal bran flakes, corn flakes or wholemeal oat flakes. Significant differences calculated with the Wilcoxon t-test. There were no significant differences between the mean incremental satiety scores.

Ingestion of cereal bran flakes or wholemeal oat flakes did not result in a significantly higher satiety compared to the reference corn flakes meal (Table 3, Figure 3).

**Table 3:** Satiety areas under the curve (AUCs) after ingestion of meals consisting of wholemeal oat flakes, cereal bran flakes or corn flakes in twelve healthy subjects^1^. Significant differences of satiety AUCs were calculated with the Wilcoxon t-test. There were no significant differences between the AUCs.

AUC	Wholemeal Oat Flakes *cm^2^*	Cereal Bran Flakes *cm^2^*	Corn Flakes *cm^2^*
0 – 5 min	14.0 ± 2.1	16.4 ± 2.4	12.5 ± 2.5
0 – 25 min	128.1 ± 18.7	148.5 ± 19.4	112.9 ± 21.1
0 – 45 min	244.0 ± 34.7	275.6 ± 34.2	206.2 ± 38.0
0 – 65 min	359.8 ± 45.8	386.4 ± 46.9	289.4 ± 52.6
0 – 85 min	459.8 ± 56.6	466.6 ± 56.1	363.5 ± 65.8
0–105 min	542.3 ± 66.1	544.9 ± 64.9	418.1 ± 75.0
0 – 125 min	602.4 ± 74.6	600.8 ± 12.9	454.6 ± 80.1

^1 ^Mean ± SEM; n = 12

## Discussion

The results of this study show that the presence of fibre in a semisolid meal does not affect total postprandial blood glucose or satiety responses in healthy subjects, despite the delay in gastric emptying for the product containing the higher amount of fibre (cereal bran flakes). This study was designed to evaluate the effect of commercial cereals on blood glucose, satiety and GER. The postprandial glucose response was reduced at the initial postprandial phase after the cereal bran flakes meal compared to the corn flakes meal. Similar results have previously been presented showing a lower early postprandial blood glucose response after the intake of cereal bran flakes when compared to corn flakes [30]. In the same study it was shown that this lower postprandial blood glucose response was related to a higher initial postprandial plasma insulin response after a meal composed of 119.2 g cereal bran flakes compared to a meal of 60.9 g corn flakes [30]. However, the total postprandial insulin AUC was identical for the two meals and gastric emptying was not measured [30]. It is obvious that healthy subjects have a sufficient insulin response, thus giving a normal blood glucose response. Consequently, we have had similar total postprandial blood glucose AUCs for the products in our study. Moreover, the cereal bran flakes meal had a smaller amount of carbohydrates than that of the corn flakes meal. However, if we had used the same amount of carbohydrates from each cereal brand in our study, we would have had a larger difference in energy, which, in turn, could potentially have influenced the GER results. An increased caloric value of a meal can delay the GER [31]. The cereal bran flakes meal had the lowest total caloric value, 298 kcal, compared to the other meals with 320 kcal, respectively. Still, the difference in GER was only significant between the cereal bran flakes meal and the wholemeal oat flakes, probably due to the higher amount of fibre in the cereal bran flakes meal. Also, the glucose response was reduced at the end of the postprandial phase after the cereal bran flakes meal compared to the wholemeal oat flakes meal (Figure 1), which could be related to the lower GER (Figure 3). However, in patients with type 2 diabetes, oat bran flour containing 9.4 g β-glucan lowered the postprandial glycemia [24]. In the same study on type 2 diabetes patients using oat bran crisps containing 3.0 g β-glucan, the postprandial glycemia was also reduced, although the reduction was only half as large as compared to oat bran flour containing 9.4 g β-glucan [24]. It has previously been shown in type 2 diabetes patients that each gram of β-glucan in food can lower the GI by four GI units [23]. The wholemeal oat flakes meal contained only 0.5 g β-glucan. Probably the amount of β-glucan was too small to affect the blood glucose response. In this study we could not show any significant difference in satiety despite a delayed gastric emptying after the cereal bran flakes meal compared to the wholemeal oat flakes meal. A delay in GER has previously been shown to increase satiety [32]. However, despite a difference in postprandial blood glucose and satiety hormones – such as ghrelin and plasma peptide YY (PYY) – after consumption of oat and wheat fibre, no difference was found with regard to satiety in healthy subjects [33].

The American Diabetes Association (ADA) recommends a daily intake of 14 g fibre/1.000 kcal and foods with whole grains to prevent diabetes [1]. The intake of total dietary fibre, particularly insoluble and cereal fibre, has been shown to have an inverse association with diabetes type 2 [2]. Insoluble fibre and fibre from fruit, vegetables, or legumes have been shown to be unrelated to diabetes [2]. It is unclear whether low glycemic index food prevents diabetes mellitus. Still, the ADA recommends low glycemic index foods that are rich in fibre [1]. However, the composition of the commercial product should be more important than the fibre content alone. Several studies have shown that there was no difference in postprandial blood glucose response directly after the intake of fibre, whereas a beneficial effect on glucose metabolism was observed on the second meal test due to colonic fermentation [14,34,35].

## Conclusion

The result of this study demonstrates that the intake of equal amounts of either cereal bran flakes and wholemeal oat flakes has no effect on the total postprandial blood glucose response or satiety when compared to corn flakes. Furthermore, this study shows that the intake of cereal bran flakes slows the GER when compared to wholemeal oat flakes and corn flakes, probably due to a higher content of fibre. Since these products do not differ in terms of glucose response and satiety on healthy subjects, they should be considered equivalent in this respect. However, this study does not exclude a potential difference between the meals with regard to a delayed second meal effect.

## Competing interests

The author(s) declare that they have no competing interests.

## Authors' contributions

JH participated in the design of the study, performed the statistical calculations and the graphs and drafted the manuscript. RF and JW participated in the design of the study, recruited subjects, collected the data and drafted the manuscript. GD participated in the design of the study, performed the statistical calculations and the graphs, paid for the study and participated in drafting the manuscript. OB participated in the design of the study and performed the ultrasound examinations. LOA participated in the design of the study and in drafting the manuscript. All authors read and approved the final manuscript. All authors lacked any conflict of interest.
